# Assessing and Supporting Adolescents' Capacity for Autonomous Decision-Making in Health-Care Settings: New Guidance From the World Health Organization

**DOI:** 10.1016/j.jadohealth.2022.04.005

**Published:** 2022-07

**Authors:** Valentina Baltag, Yusuke Takeuchi, Regina Guthold, Anne-Emmanuelle Ambresin

**Affiliations:** a Department of Maternal, Newborn, Child & Adolescent Health & Ageing, World Health Organization, Geneva, Switzerland; b Interdisciplinary Division for Adolescent Health (DISA), Lausanne University Hospital (CHUV), Lausanne, Switzerland; c Faculty of Biology and Medicine, University of Lausanne (UNIL), Lausanne, Switzerland

**Figure 1:**
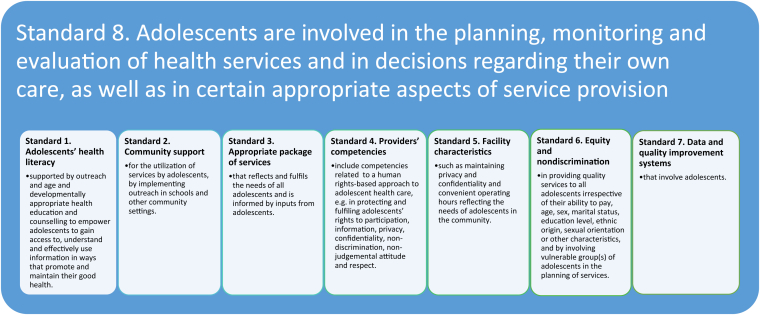
Adolescent participation in Global Standards for quality health-care services for adolescents.

Source: adapted from Global Standards for quality health-care services for adolescents, WHO 2015.

In 2015, the World Health Organization published Global Standards for quality health-care services for adolescents—defined by WHO as persons 10–19 years old—to support countries to transform how health systems and services respond to the health needs of adolescents [1]. The notion of adolescent participation is central to the essential elements of adolescent-responsive health services as described in eight Global Standards. It informs Standard 8, which explicitly emphasizes the importance of adolescent participation in decisions regarding their own care and is a prerequisite for a successful implementation of other standards (Figure 1).

Recently, WHO has published for the first time a guidance that describes a step-by-step process in assessing and supporting adolescents' capacity for autonomous decision-making [2]. Adolescents' right to participate in decisions that affect their lives is enshrined in the Convention on the Rights of the Child [3] that stipulates the right of the child who is capable of forming his or her own views to express those views freely in all matters affecting him or her and having those views given due weight in accordance with the age and maturity [4]. Central therefore to the application of this right and to the implementation of Global Standards is the recognition of the evolving nature of adolescent capacity to understand matters affecting his or her life and health. The more an adolescent “knows, has experienced and understands, the more the parent, legal guardian, or other persons legally responsible for him or her can transform direction and guidance into reminders and advice and later into exchange on an equal footing [5]”.

Application of the framework of legal rights to clinical situations may be inherently problematic. In clinical settings, a significant challenge is reconciling evolving capacity during developmental changes in adolescence with concrete evaluation of decision-making capacity at a specific time for a specific situation. It is important to differentiate competence from capacity: “competence” is a legal concept that refers to the right to provide an opinion or make an autonomous decision (e.g., the right to sign an informed consent for health service); “capacity” is a clinical concept that refers to individual psychological and cognitive ability to understand information, reason, and reflect in order to make a decision [6].

**Figure 2:**
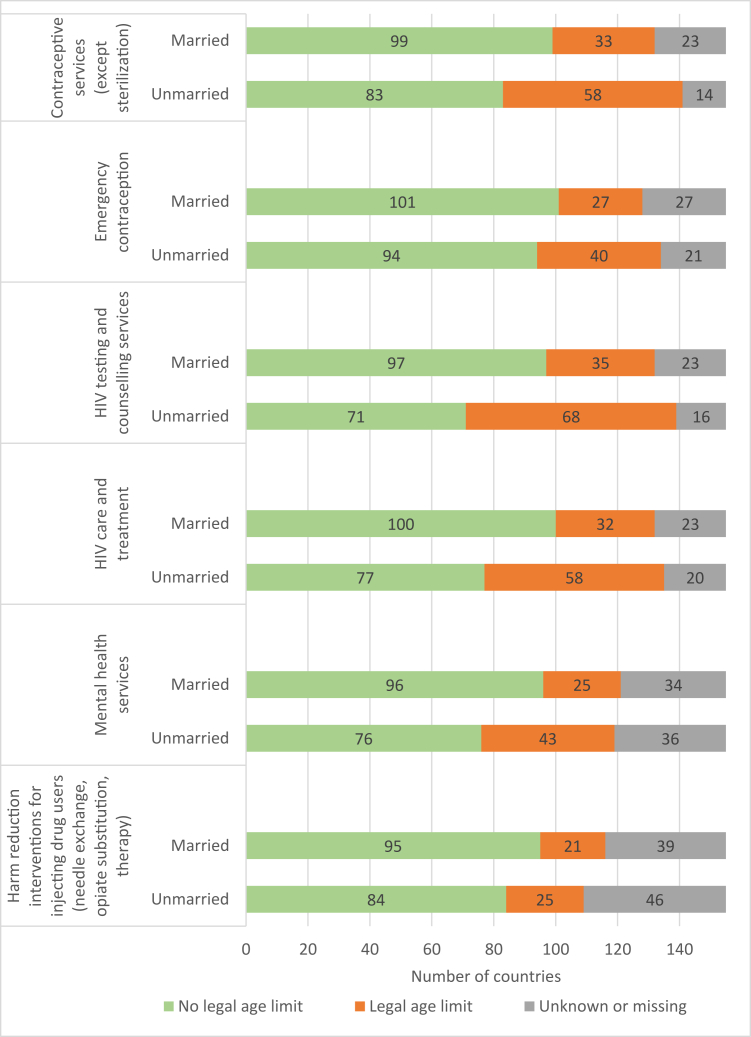
Number of countries without or with a legal age limit for married and unmarried adolescents to provide consent, without parental/legal guardian or spousal consent, to selected services.

Source: Sexual, Reproductive, Maternal, Newborn, Child and Adolescent Health Policy Survey 2018-2019 [8].

National legal frameworks are highly heterogeneous [7]. Often age limits for adolescents to provide consent vary by marital status and by the type of health service [8]. A greater number of countries have legal age limits for unmarried adolescents as compared to married adolescents. This makes it more difficult for unmarried adolescents to access services, particularly HIV as well as mental health services Figure 2).

“Minors” is a legal term that refers to adolescents who are under the age of majority, whereas the term “adolescents” does not have a legal definition. Some countries or states have defined in law the age at which minor adolescents are allowed to make decisions about their health, but age limits are not defined by law in most countries. Thus, the task of determining their competence is often left to health-care providers and their teams. Where rigid age limits exist, they have been a suboptimal measure of adolescent capacity because they do not account for the differences in adolescents' developmental trajectories nor do they account for the diversity of concrete clinical situations and personal circumstances or states of mind (e.g., hot cognition) at a given moment.

Therefore, in the Accelerated Action for the Health of Adolescents, WHO recommends removing parent consent requirements for counseling and advice services; adopting flexible policies to allow specific groups of adolescents to be considered “mature minors”; and, as much as possible, allowing autonomous decisions regarding their own health to be made on a case-by-case basis, informed by the adolescent's developmental stage, as well as careful evaluation of risks, security, and other potentially influential factors in the local context [9,10]. The evolving nature of capacity, described above, implies the recognition of the developmental differences between younger adolescents (e.g., age 12 or 13) and older adolescents (e.g., age 17 and above) as well as the significant role that parents and other supportive adults may play for adolescents.

Until now, there has not been any practical guidance to health-care providers and their teams on how to assess and support adolescents in making decisions about their health. The newly published guidance from WHO has four key features that are described below:

First, as the title suggests, the guidance is not only about assessing but also supporting and enhancing adolescent capacity. It is based on the principles of shared decision-making and people-centered care from the perspectives of individuals, their family, and their community. This is an important issue in adolescent health care, because adolescents sometimes do not share the view of the health professional or their parents or legal guardian, not because of a lack of discernment but because of different values. Yet, in some situations, adolescents lack the understanding and capacity to make free, informed, autonomous decisions and require support from adults. Therefore, health-care providers sometimes have to make decisions to protect the adolescent's well-being, even against their will, while still bearing in mind the need to support adolescents' autonomy to make their own decisions. The tool supports the navigation of the ethical dilemma between protection and autonomy underpinning adolescent health care and helps avoid the risk of a paternalistic approach of making decisions for adolescents “for their good.”

**Figure 3:**
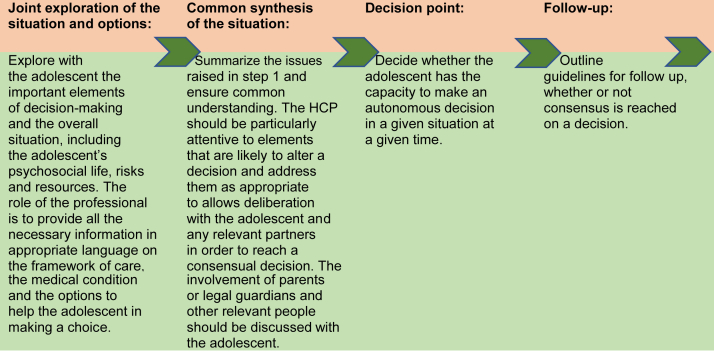
Practical steps for assessing and supporting adolescents' capacity for autonomous decision-making.

Second, the guidance includes elements of assessment of cognitive capacity for decision-making and adds a broader evaluation of risks and resources and the emotional state of adolescents, all of which influence their decision-making capacity (Figure 3).

Third, although the process has four steps, it is not designed as a rigidly linear process. An integrated, dynamic approach is proposed, with reiteration of the different steps as necessary.

Fourth, the tool promotes the application of the principles of medical ethics—beneficence, nonmaleficence, respect (autonomy), and justice—based on the context of each situation. Often two or more ethical principles may conflict, and because no principle is more important than another, they do not always provide answers to all the questions or conflicts that may arise. When principles conflict, the guide proposes a deliberative balancing that may provide reasons for considering one value more important than another in a given situation. The tool therefore integrates the perspective of rights, ethics, context, social determinants, and vulnerability into the decision-making process.

The WHO guide represents the results of over two years work by WHO and a group of 13 international experts representing different contexts and geographical regions of the world gathering expertise in pediatrics and adolescent health care, children's rights, bioethics, developmental psychology, and research in competence and decision-making capacity. The tool provides a practical support for a rights-based approach to adolescent health care. It will contribute to enhancing providers' competencies in adolescent-responsive health care and ultimately will advance the goals of universal health coverage with quality services for the world’s 1.2 billion adolescents.
